# Inhibition of erythropoietin signalling destroys xenografts of ovarian and uterine cancers in nude mice

**DOI:** 10.1054/bjoc.2000.1666

**Published:** 2001-03

**Authors:** Y Yasuda, T Musha, H Tanaka, Y Fujita, H Fujita, H Utsumi, T Matsuo, S Masuda, M Nagao, R Sasaki, Y Nakamura

**Affiliations:** Department of Anatomy, Kinki University School of Medicine, Osakasayama, 589–8511, Japan; Department of Obstetrics and Gynecology, Kyorin University School of Medicine, Mitaka, 181-8611, Japan; Laboratory of Environmental Biology, Hokkaido University, Sapporo, 060-8648, Japan; Research Reactor Institute, Kyoto University, Osaka, 590-0451, Japan; Laboratory of Biosignals and Response, Graduate School of Biostudies, Kyoto University, Kyoto, 606–8507, Japan

**Keywords:** erythropoietin mRNA, erythropoietin receptor mRNA, malignant cells, capillary, apoptosis

## Abstract

We have recently shown that malignant tumours from the ovary and uterus expressed erythropoietin (Epo) and its receptor (EpoR), and that deprivation of Epo signal in tumour blocks induced death of malignant cells and capillary endothelial cells in vitro (Yasuda et al, submitted). These in vitro results prompted us to examine the effect of Epo-signal withdrawal on tumours in vivo. RT-PCR analysis demonstrated the expression of mRNAs for Epo and EpoR in the transplants of uterine and ovarian tumours in nude mice. Then we injected locally anti-Epo antibody or soluble form of EpoR into the transplants. At 12 h, 1, 7 or 14 days after the injection, all transplants were resected and examined macro- and microscopically. Tumour size was reduced in Epo signal-deprived transplants. Immunohistochemical examinations revealed destruction of Epo-responding malignant and capillary endothelial cells through apoptotic death. The degree of tumour regression correlated well with the dose and frequency of the injections. Control xenografts with saline injection or needle insertion showed well-developed tumour masses. This Epo response pathway will have profound implications for our understanding of the development and progression of malignant tumours and for the use of Epo-signal deprivation as an effective therapy. © 2001 Cancer Research Campaign http://www.bjcancer.com


British Journal of Cancer (2001) 84(6), 836–843
© 2001 Cancer Research Campaign
doi: 10.1054/ bjoc.2000.1666, available online at http://www.idealibrary.com on IDEAL®

http://www.bjcancer.com

### Inhibition of erythropoietin signalling destroys xenografts of ovarian and uterine cancers in nude mice

**Y Yasuda[1], T Musha[2], H Tanaka[2], Y Fujita[1], H Fujita[3], H Utsumi[4], T Matsuo[1], S Masuda[5], M Nagao[5], R Sasaki[5] and Y Nakamura[2]**

[1]Department of Anatomy, Kinki University School of Medicine, Osakasayama, 589–8511, Japan; [2]Department of Obstetrics and Gynecology, Kyorin University School of Medicine, Mitaka, 181-8611, Japan; [3]Laboratory of Environmental Biology, Hokkaido University, Sapporo, 060-8648, Japan; [4]Research Reactor Institute, Kyoto University, Osaka, 590-0451, Japan; [5]Laboratory of Biosignals and Response, Graduate School of Biostudies, Kyoto University, Kyoto, 606–8507, Japan

**Summary** We have recently shown that malignant tumours from the ovary and uterus expressed erythropoietin (Epo) and its receptor (EpoR), and that deprivation of Epo signal in tumour blocks induced death of malignant cells and capillary endothelial cells in vitro (Yasuda et al, submitted). These in vitro results prompted us to examine the effect of Epo-signal withdrawal on tumours in vivo. RT-PCR analysis demonstrated the expression of mRNAs for Epo and EpoR in the transplants of uterine and ovarian tumours in nude mice. Then we injected locally anti-Epo antibody or soluble form of EpoR into the transplants. At 12 h, 1, 7 or 14 days after the injection, all transplants were resected and examined macro- and microscopically. Tumour size was reduced in Epo signal-deprived transplants. Immunohistochemical examinations revealed destruction of Epo-responding malignant and capillary endothelial cells through apoptotic death. The degree of tumour regression correlated well with the dose and frequency of the injections. Control xenografts with saline injection or needle insertion showed well-developed tumour masses. This Epo response pathway will have profound implications for our understanding of the development and progression of malignant tumours and for the use of Epo-signal deprivation as an effective therapy. © 2001 Cancer Research Campaign http://www.bjcancer.com

**Keywords**: erythropoietin mRNA; erythropoietin receptor mRNA; malignant cells; capillary; apoptosis

Erythropoietin (Epo) from the kidney and liver is involved in a well-recognized function, erythropoiesis (Kranz, 1991; Jelkman, 1992; Youssoufian et al, 1993; Wu et al, 1995; Lin et al, 1996; Masuda et al, 1999). Epo supports the survival of erythroid precursor cells and stimulates their proliferation and differentiation by binding to its specific receptor (EpoR), which belongs to a family of cytokine receptors that have no tyrosine kinase domain. The mitogenic action of Epo appears to be caused by Epo-induced homodimerization of EpoR and the subsequent activation of JAK2 through tyrosine phosphorylation, ultimately leading to activation of a transcriptional factor, STAT5 (Youssoufian et al, 1993; Ihle, 1995). Recently, Epo has been shown to be produced in the brain by astrocytes and to protect neurons from ischaemic damage via EpoR expressed in neurons (Sadamoto et al, 1998; Sakanaka et al, 1998). The neuroprotective action of Epo is manifested by a reduction of nitric oxide neurotoxicity (Sakanaka et al, 1998) but its precise mechanism is not known. Production of Epo at these 3 sites (kidney, liver and brain) is hypoxia-inducible (Porter and Goldberg, 1993; Bunn and Poyton, 1996; Guillemin and Krasnow, 1997; Wenger and Gassmann, 1997; Ratcliffe et al, 1998; Semenza, 1998).

Vascular endothelial cells express EpoR and respond to Epo in vitro. EpoR mRNA is expressed in endothelial cells from various sources (Anagnostou et al, 1994; Yamaji et al, 1996). Epo stimulates proliferation and migration of endothelial cells (Anagnostou et al, 1990) and also in vitro angiogenesis (Carlini et al, 1995). Epo signalling as a mitogen of endothelial cells is conducted via tyrosine phosphorylation of proteins including phosphorylation of transcription factor STAT5, which is similar to that occurring in erythroid cells (Haller et al, 1996). More recently Epo has been shown to induce a potent angiogenic response in the chick chorioallantoic membrane (Ribatti et al, 1999). We have presented in vivo evidence that Epo plays a critical role in mouse uterine angiogenesis via EpoR expressed in vascular endothelial cells of the uterine endometrium (Yasuda et al, 1998). A new site for Epo production exists in the uterus where Epo production is induced by 17β-estradiol (E2) (Yasuda et al, 1998). In contrast to that in the liver, kidney and brain, Epo production by the uterus is not hypoxia-inducible in the absence of E2 but it gains hypoxia-inducibility in the presence of E2. We have also found that the mouse ovary expresses Epo mRNA (Yasuda et al, 1998).

On the basis of the finding that female reproductive tissues produce Epo, we speculated that Epo signalling might contribute to the development and the progression of malignant tumours of these organs. Previously we reported that all malignant tumour specimens derived from the ovary and uterus expressed Epo mRNA and EpoR. The cells producing Epo mRNA were not identified, but EpoR was expressed in both malignant cells and vascular endothelial cells. Withdrawal of Epo signalling through injection of anti-Epo antibody or a soluble form of EpoR (sEpoR), both of which are capable of binding with Epo, into surgically resected tumour blocks induced apoptotic death of malignant cells and regression of vessels in vitro, suggesting that Epo, in addition to its direct mitogenic effects on malignant cells, may facilitate tumour growth via an increase in the vascular supply. Encouraged by these in vitro results, we studied the effects of Epo-signal withdrawal on tumours in vivo. Here we show that the blockade of Epo signalling in xenografts of uterine and ovarian tumours in nude mice results in clearance of malignant cells and feeding vessels,

*Received 9 August 2000*
*Revised 10 November 2000*
*Accepted 23 November 2000*

*Correspondence to:* Y Yasuda

The experiments described in this paper were performed according to UKCCR Guidelines for the Welfare of Animals in Experimental Neoplasia (Second Edition).

suggesting that Epo-signal interception may be a novel therapy for malignant tumours of female reproductive organs.

### MATERIALS AND METHODS

### Animals

Hairless female mice (Balb/c, Jcl-nu) were purchased from Clea Inc, Japan, at 5 weeks of age, placed one by one in aseptic chambers and given sterilized pellets and water ad libitum in a specific pathogen-free room. At 6 weeks of age or older, they were inoculated with a piece of cancer specimen or transplant.

### Transplantation

Resected tumour specimens were put into α-MEM (Gibco) with 10% fetal calf serum containing streptomycin (100 µ1 $ml^{-1}$) and penicillin (100 IU $ml^{-1}$) at 4˚C and cut into small pieces. The patients gave informed consent for these specimens to be used for the experiments. Transplants were derived from 14 primary adenocarcinomas (ADC) and 3 squamous cell carcinomas (SCC) of the cervix, and 2 ovarian ADC; secondary tumours were from 1 primary ovarian ADC.

Under deep anaesthesia an incision was made in the intrascapular region of the mouse and a piece of primary or secondary tumour measuring approximately 5 × 5 × 5 mm was inserted subcutaneously. The incision was sutured and sprayed with Novactan. The length and width of the implants were measured 3 times a week. Tumour growth was calculated according to the Battle Memorial Institute Protocol (Ovejara et al, 1978).

### RT-PCR amplification

To confirm the expression of mRNAs for Epo and EpoR in the xenografts, 5 tumours from ovarian and uterine ADC or SCC were transplanted. When their diameter had reached 12–15 mm, they were extirpated and frozen in liquid nitrogen. The frozen materials were homogenized to extract total RNA.

Total RNA was prepared with the RNA Extraction kit (Amersham). The RT reaction was performed with an avian myeloblastosis virus (AMV) reverse transcriptase, random nanomer primer (Takara) and 1 µg of each RNA in a total volume of 20 µl with the use of a TaKaRa RNA LAPCR™ Kit (AMV) Ver. 1.1. PCR primer of Epo, EpoR and β-actin were those described previously (Yasuda et al, 1993). PCR cycles and conditions for denaturation, annealing, and elongation were 28 cycles, 30 at 94˚C, 30 s at 60˚C, and 1.5 min at 72˚C of Epo; 30 cycles, 1 min at 94˚C, 2 min at 55˚C and 3 min at 72˚C of EpoR and β-actin. The amplified DNA was fractionated by electrophoresis on a 12% agarose gel (BioProduct) and stained with ethidium bromide.

### Treatment

#### *Injection materials*

Anti-Epo monoclonal antibody, R2, a gift from the Snow Brand Milk Product Co. (Tokyo, Japan) (Goto et al, 1989), and sEpoR prepared by the methods of Nagao et al (1992) were dissolved in saline (Ohtsuka, Tokyo, Japan). Saline was used as the control solution. To these solutions Evans blue dye (Merck, Darmstadt, Germany) was added to make a final concentration of 0.025% for colouring.

#### *Injection*

When the transplanted tumours had grown larger than 3 times their initial size, 0.2 ml of R2 or sEpoR was injected 3 or 4 times at intervals of 60 min, 6 h or 12 h into each tumour (Table 1). In some control transplants a needle was inserted at the same intervals. The type of transplant, concentrations of R2 or sEpoR and injection schedule are listed in Table 1.

### Macroscopy

At 12 hours or 1, 7 and 14 days after treatment, the tumour masses were resected under deep anaesthesia. After examination under a dissecting microscope, the tumours were fixed in Zamboni solution and processed for immunostaining.

**Table 1** Regression of uterine and ovarian tumours transplanted in nude mice injected with anti-Epo antibody or sEpoR

| Experiment | Type of tumour transplanted (age of patient) | No. of mice transplanted | Treatment schedule[a] | | | Extirpation | Tumour size at extirpation (%)[b] |
|---|---|---|---|---|---|---|---|
| | | | Injected | Concn. mg $ml^{-1}$ | Injection time (h) | After last injection (day) | |
| 1 | ADC-endometrium | 1 | R2 | 0.125 | 0, 6, 12 | 1 | 66 |
| 2 | (57) | 1 | R2 | 0.25 | 0, 6, 12 | 1 | 63 |
| 3 | ADC-endometrium | 2 | R2 | 8 | 0, 1, 2 | 0.5 | 61 |
| 4 | (64) | 2 | R2 | 16 | 0, 1, 2 | 0.5 | 34 |
| 5 | SCC-cervix (69) | 3 | R2 | 16 | 0, 1, 2 | 1 | 17 |
| 6 | ADC-cervix (38) | 3 | sEpoR | 0.2 | 0, 1, 2 | 1 | 65 |
| 7 | ADC-ovary secondary | 2 | sEpoR | 0.2 | 0, 1, 2, 3 | 7 | 48 |
| 8 | ADC-ovary primary (78) | 2 | R2 | 8 | 0, 1, 2, 14 | 14 | 23[c], 11 |

[a]When the transplanted tumours grew larger than 3 times their initial size, anti-Epo monoclonal antibody (R2) or soluble form of EpoR (sEpoR) was injected according to the treatment schedule indicated. Saline was injected in the control mice bearing the transplanted tumours in the same schedule. [b]Tumour size relative to that just before the injection. When two or three mice were transplanted with individual tumours (Exps 3–8), average tumour sizes are shown. [c]Tumour size 7 days after injection.

© 2001 Cancer Research Campaign 

### Immunohistochemistry

After cryoprotection, the specimens were cut in 7 µm slices on a cryostat (Leica, Germany) and processed for immunostaining as described previously (Yasuda et al, 1992). The following antibodies were used as the primary antibodies; anti-EpoR (Yasuda et al, 1992) (1/500 dilution), anti Factor-VIII (FVIII) (DAKO) (1/200 dilution), and anti-mouse macrophages (F4/80, Serotec) (1/200 dilution). The staining specificity of EpoR antibody was confirmed with the use of the antibody preabsorbed with sEpoR. No antigens were available for FVIII and F4/80, so rabbit IgG (1/200 dilution) (Cappel) and rat IgG (1/200 dilution) (Zymed) were used, respectively. To detect apoptosis, TdT assay was done with a TdT assay kit (Oncor, Gaitherberg, MD). The positive control for TdT assay was confirmed on sections of adult rat testis, and the negative control was performed on sections of rat testis without TdT enzyme (data not shown). In all tumours, 15 to 20 sections from 3 to 5 thick slices of each specimen were stained with each antibody, and 4 to 6 sections from a tumour were used for TdT assay. The pathological features in each specimen were graded in sections stained with each antibody or subjected to TdT assay; they are summarized in Table 2.

### Capillary counting

Sections of all specimens were stained with anti-FVIII antiserum. Cross- and longitudinally sectioned capillaries were counted with an eye-piece ocular micrometer in 100 areas of each specimen ($2.85 \times 10^{-2}$ $mm^2$) in sections separated by 21 µm.

### RESULTS

### Transplant

A total of 45 cancer fragments were implanted, and 36 primary and secondary transplants grew successfully. One of the ovarian xenografts grew large enough to be retransplanted successfully. It was extirpated, cut into fragments, and implanted into 5 other nude mice 4.5 months after the first implantation. One transplant became necrotic at 5 months and was excluded from the experimental procedures.

### Expression of Epo and EpoR mRNA

5 transplants derived from 1 cervical SCC, 3 endometrial ADC (ADCE) and one ovarian ADC (ADCO) were examined with RT-PCR, and the results are shown in Figure 1. Xenograft of SCC-59

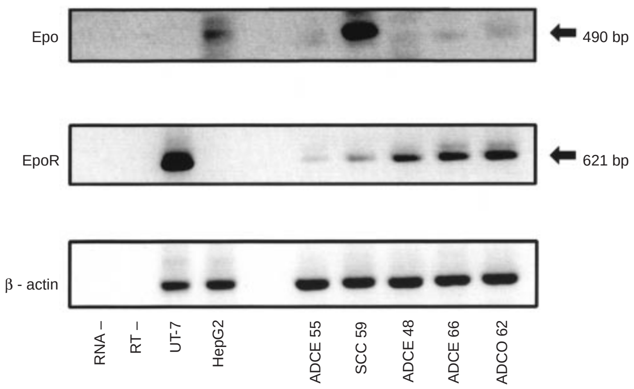

**Figure 1** Detection of Epo- and EpoR-transcript in xenografts of ovarian and uterine cancer by RT-PCR. All xenografts express Epo and EpoR mRNAs. ADCE, ADCO and SCC: origin of transplants; endometrial, ovarian ADC and squamous cell carcinoma. The number after the abbreviation means age of a patient. RNA—: no RNA; RT—: no reverse transcriptase; UT-7: megakaryoblast cell line; HepG2: hepatoma cell line

**Table 2** Pathological features in xenografts injected with R2, sEpoR or saline or receiving needle insertion

| Experiment[a] (type of tumour, injection, mg ml$^{-1}$) | | No. of tumours examined | Destroyed foci | Intact malignant foci | Endothelial degeneration | Apoptosis | Phagocyte infiltration |
|---|---|---|---|---|---|---|---|
| Uterus | 1 (ADC-E, R2, 0.125) | 1 | +2 | +1 | +1 | +2 | +2 |
| | 2 (ADC-E, R2, 0.25) | 1 | +3 | +1 | +2 | +2 | +3 |
| | (ADC-E, saline) | 1 | +1 | +2, +3 | – | +1 | +1 |
| | 3 (ADC-E, R2, 8) | 2 | +5, +5 | –, – | +3,+3 | +4, +5 | +5,+5 |
| | 4 (ADC-E, R2, 16) | 2 | +7,+5 | –, – | +5,+5 | +5, +5 | +5, +5 |
| | (ADC-E, saline) | 2 | +1, +2 | +3, +3 | –, – | +1, +1 | +2, +2 |
| | (ADC-E, needle) | 1 | +1 | +3 | – | +2 | +2 |
| | 5 (SCC-C, R2, 16) | 3 | +7, +7, +7 | –,–,– | +7, +7, +7 | +5, +5, +5 | +5, +5, +5 |
| | (SCC-C, saline) | 3 | +2, +2, +2 | +3, +2, +3 | –, –,– | –, +2, +1 | +1, +1, +1 |
| | (SCC-C, needle) | 1 | +1 | +2 | – | +1 | +1 |
| | 6 (ADC-C, sEpoR, 0.2) | 3 | +7, +7, +7 | –,–,– | +7, +7, +5 | +5, +5, +5 | +3, +3, +3 |
| | (ADC-C, saline) | 2 | +1, +1 | +5, +3 | –,– | +2, – | +2, +1 |
| Ovary | 7 (2nd, sEpoR, 0.2) | 2 | +3, +3 | +1, +1[b] | +2, +2 | +3, +3 | +5, +5 |
| | (2nd, saline) | 1 | | –[c] ·· | | | – |
| | (2nd, needle) | 1 | +1 | +5 | – | +1 | +2 |
| | 8 (ADC-O, R2, 8) | 2 | +3, +3 | +1[b], +1 | +3, +3 | +3, +3 | +5, +5 |
| | (ADC-O, saline) | 2 | +1, +2 | +5, +5 | –,– | +1,– | +3,+2 |

[a]Experimental number corresponds to that in Table 1. b, scab over the lesion, c, total necrosis. Plus (+) or minus (–) indicates presence or almost none. Number after plus means grade of each phenomenon among tumours of the same origin; +7 is most severely damaged, and +1, weakest or smallest foci; Phagocytes include neutrophils, monocytes and macrophages; ADC-C, cervical adenocarcinoma; ADC-E, endometrial adenocarcinoma; ADC-O, ovarian adenocarcinoma; SCC-C, cervical squamous cell carcinoma; 2nd, secondary ovarian tumour; R2, antibody against erythropoietin; sEpoR, soluble form of erythropoietin receptor.

*© 2001 Cancer Research Campaign*

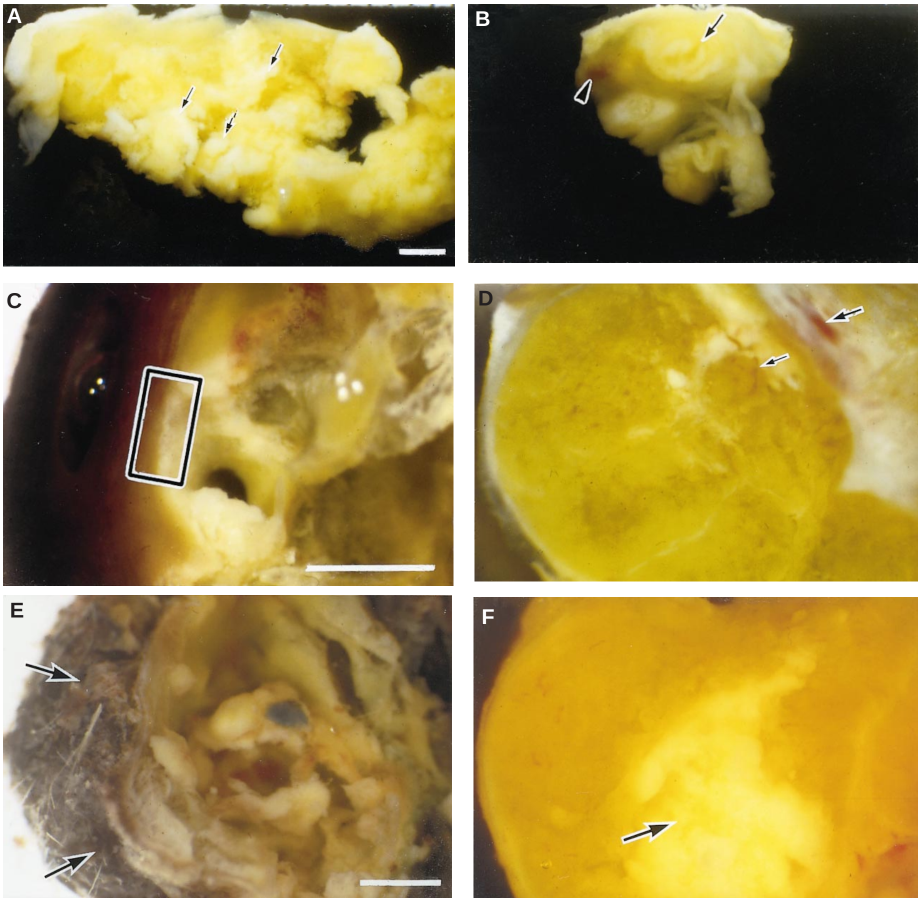

**Figure 2** Interior or cut surface of resected tumours. (**A**) Xenograft of SCC one day after injection of R2 (one of the grafts in Exp. 5 in Table 1). Note destruction of tumour masses with holes and many pale edges (arrows). (**B**) Control graft of (**A**) injected with saline (one of the controls in Exp. 5 in Table 2). Note fresh masses with central hole (arrow). Arrowhead points to vessels near the capsule. (**C**) Xenograft of primary ovarian ADC 14 days after injection of R2 (one of the grafts in Exp. 8 in Table 1). Note thick scab and large defect of tumour masses leaving fibrous tissues with many holes. (**D**) Control xenograft of (**C**) (one of the controls in Exp. 8 in Table 2). Note compact tumour masses under thin capsule and many capillaries in the tumour (small arrow) and in the capsule (large arrow). (**E**) Xenograft of secondary ovarian ADC 7 days after injection of sEpoR (one of the grafts in Exp. 7 in Table 1). Note covering scab (arrows) and destroyed tumour masses leaving membranous tissues with broken capillary twigs. Central blue spot is Evans blue dye deposit. (**F**) Control xenograft of (**E**) inserted with needle only (control in Exp. 7 in Table 2). Note compact tumour masses, central pus (arrow) and vessels. (**A**) and (**B**), (**C**), (**D**) and (**F**), are at the same magnification. Scale bar, 100 μm showed high expression of Epo mRNA, and the grafts of ADCE-55, ADCE-48, ADCE-66 and ADCO-62 showed weak expression. HepG2 is a human hepatoma cell line that produces Epo (Maxwell et al, 1993). Epo mRNA was clearly detected in HepG2. 3 transplants of ADCE-48, ADCE-66 and ADCO-62 showed strong expression of EpoR mRNA, and the 2 transplants of ADCE-55 and SCC-59 showed weak expression. UT-7 is a megakaryoblast cell line which expresses EpoR mRNA (Komatsu and Fujita, 1993). EpoR mRNA was strongly expressed in UT-7. All samples showed unchanged β-actin mRNA.

### Tumour growth

Growth curves of all grafts were plotted, and the degree of tumour regression after injection of R2 or sEpoR was calculated from each growth curve. Table 1 shows the injection schedule and the tumour regression induced by injection of R2 or sEpoR that can deplete Epo. Injection of R2 or sEpoR reduced the size of all transplanted tumours. After 12 h of treatment the tumours injected 3 times with 16 mg $ml^{-1}$ of R2 had shrunk in size to about one half of the size of those injected with 8 mg $ml^{-1}$ of R2 (Exps 3 and 4 in Table 1). On day 14 after the last injection of 8 mg $ml^{-1}$ of R2, the tumours were half the size they measured on day 7 (Exp. 8 in Table 1). The control tumours injected with saline were 93 to 95% of their original size or even larger than before injection. Needle insertion caused no regression. Therefore, the reduction of tumour size appears to be dose-dependent and also to depend on the interval since the last injection of the material disrupting Epo-signal transduction.

### Macroscopy

All 35 xenografts were examined. Typical effects of R2 or sEpoR on tumours are shown in Figure 2. At 24 h after injection of R2 compact tumour masses were difficult to detect under the tumour capsule (Figure 2A), while the control tumour had small or large compact tumour masses with or without small holes or necrotic foci (Figure 2B). The transplants at 7 or 14 days after the injection of R2 (Figure 2C) or sEpoR (Figure 2E) had few fresh compact tumour masses under the capsule beneath the scab. Some twigs of capillaries were detectable in tumours injected with sEpoR (Figure 2E) and almost all twigs disappeared in those injected with R2

*© 2001 Cancer Research Campaign* 

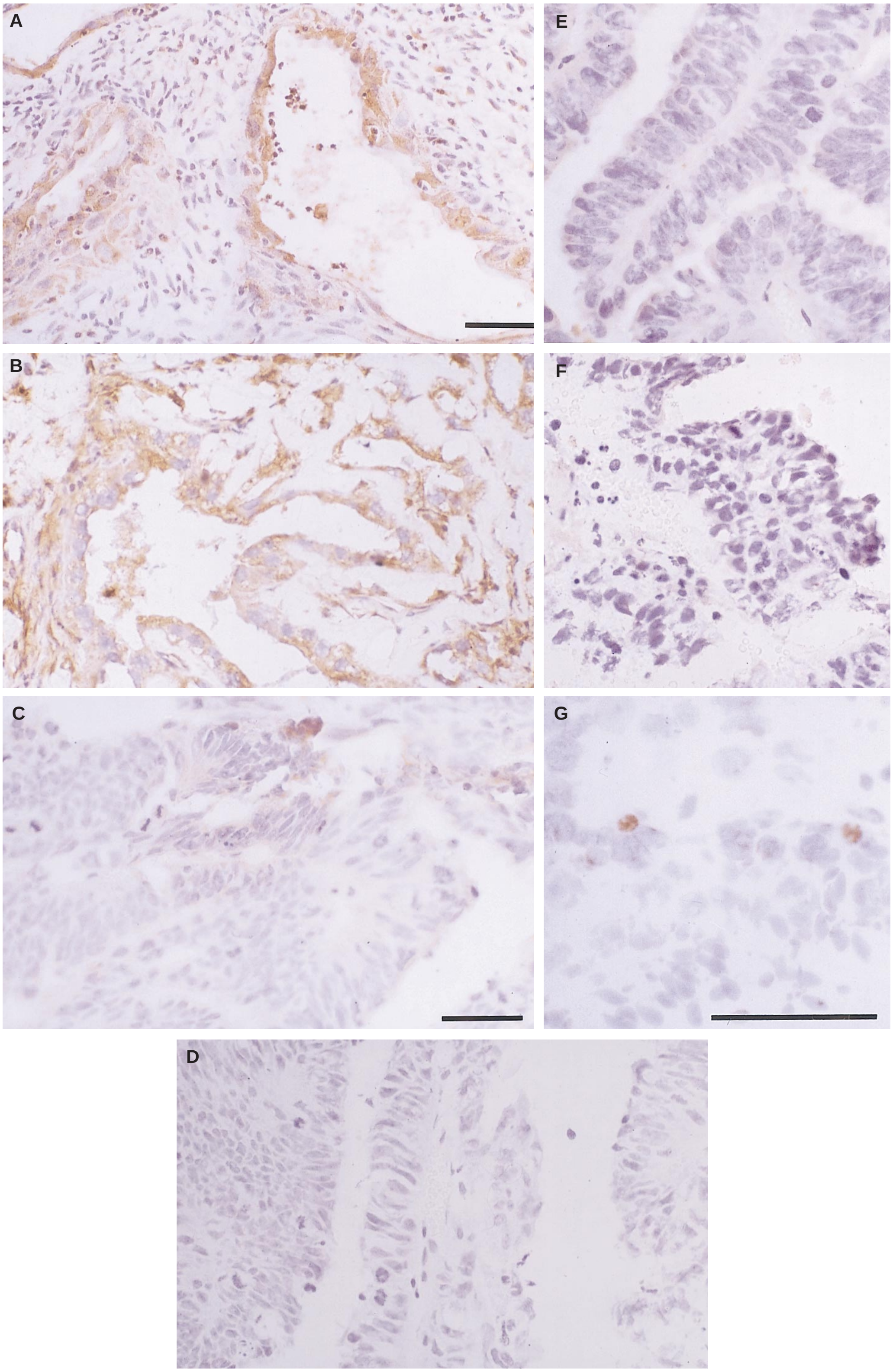

**Figure 3** Localization of EpoR in xenografts and the staining specificity of each antibody. (**A–C**) were stained with anti-EpoR; (**D**), with EpoR antiserum preabsorbed with sEpoR; (**E**), with rabbit IgG; (**F**), with rat IgG; (**G**), adapted to TdT assay. (**A**) Xenograft of ADC-E (Exp. 4 in Table 2). Note immunoreactive EpoR in degenerating glandular epithelium (arrows), and recruitment of neutrophils (small arrows). (**B**) Control of (**A**) with needle insertions. Note immunoreactive EpoR in well-developed glandular epithelium (arrows) and vascular endothelium (small arrows). (**C**) Xenograft of ADC-O (control of Exp. 7 in Table 2) shows immunoreactive malignant cells (arrows) and capillary endothelium (small arrows). (**D**) Another sections shown in (**C**). No immunoreactive EpoR is discernible. (**E**) Xenograft of ADC-O shown in Figure 2 (**D**). Note no positive stainability in vascular endothelium (arrows). (**F**) Xenograft of ADC-O shown in Figure 2 (**E**). Note no stainable monocytes and macrophages (arrows). (**G**) Rat testis. Note positive reaction in spermatogonia (arrows). (**A**) and (**B**), and (**C–F**) are at the same magnification. Scale bar, 50 μm

*© 2001 Cancer Research Campaign*

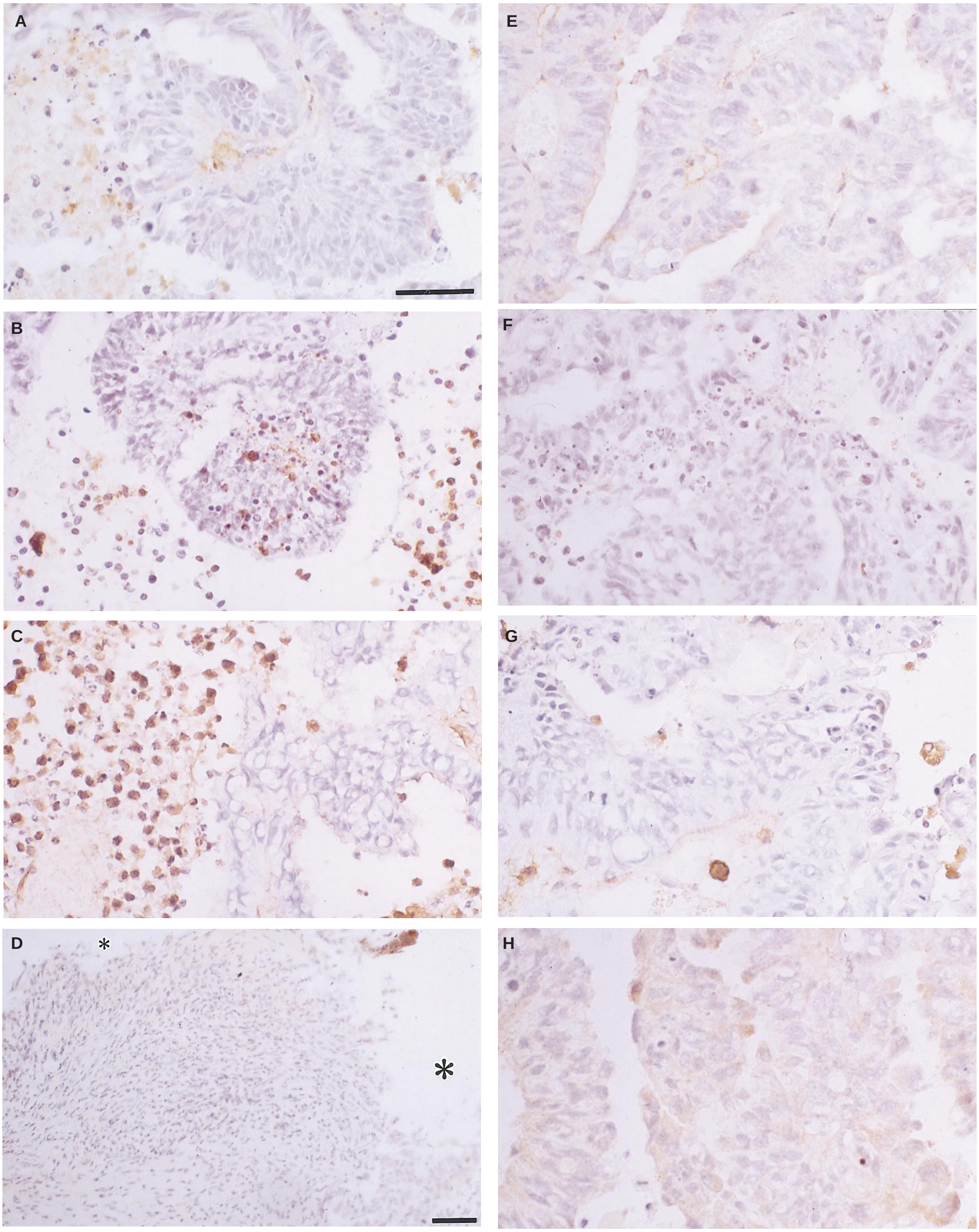

**Figure 4** Destructive processes of xenografts of ovarian tumour. (**A–C**): xenografts of Exp. 7 in Table 2; (**E–G**) and (**H**): xenograft of control of Exp. 8 in Table 2. (**A**) and (**D**) were stained with anti-FVIII; (**B**) and (**F**), subjected to TdT assay; (**C**) and (**G**), with anti-F4/80; (**D**) and (**H**), with anti- EpoR antibody. (**A**) Shows destruction of malignant tissues in left half containing small or large mononuclear cells (arrows), and a few capillaries (small arrows). (**B**) Note many apoptotic cells (arrows) in both malignant tissues including endothelial cell (smallest arrow) and destructive regions (small arrows). (**C**) Note many monocytes and macrophages (arrows) and fragmented nuclei (small arrows). (**E**) Shows well-developed vascular nets. Arrows point to endothelial cells. (**F**) Note less numerous apoptotic cells than in (**B**). (**G**) Note sporadic distribution of macrophages (arrows). (**D**) Xenograft (Exp. 8 in Table 2) shows little malignant spots (arrows) surrounded with fibrous connective tissues. Note strong EpoR immunoreactivity of dying malignant cells (small arrows) in corresponding rectangle shown in Figure 2 (**C**). The scab covering (asterisks) was melted away during staining procedures. (**H**) Control of (**D**). Note malignant tissues with EpoR immunoreactivity. All panels except (**D**) are at the same magnification. Scale bar, 50 μm

*© 2001 Cancer Research Campaign* 

(Figure 2C). The control tumours that received saline injection (Figure 2D) or needle insertion (Figure 2F) had compact fresh tumour masses with vessels or pus-like material in the middle of the tumour (Figure 2F).

### Microscopy

The grafts injected with high or low doses of R2 or sEpoR showed the destruction of characteristic malignant structures to various degrees (Table 2), and such alterations were detectable 12 h after injection (Figure 3A): in the grafts of ADCE (Exp. 4 in Tables 1 and 2) the glandular epithelial cells that showed positive EpoR-immunoreactivity were destroyed, then invaded with neutrophils leading to disappearance of glandular epithelial cells (Figure 3A). The needled control graft (control of Exp. 4 in Table 2) showed well-developed large or small glandular structures in which EpoR-immunoreactivity was detectable in glandular epithelial and vascular endothelial cells (Figure 3B). 7 days after needle-insertion, the graft (control of Exp. 7 in Table 2) still had well-developed malignant tissues expressing EpoR immunoreactivity (Figure 3C). The staining specificity of the EpoR antibody was confirmed in other sections shown in Figure 3C (Figure 3D). The staining specificity for FVIII and F4/80 antibody was confirmed in the sections of each graft, control and Exp. 8 in Table 2, respectively and is shown in Figures 3 E and 3 F. The positive control for TdT assay was confirmed in the spermatogonia of the rat testis (Figure 3G).

The grafts injected with sEpoR (Exp. 7 in Table 2) showed severe destruction of malignant foci with fewer vascular nets (Figure 4A), many more neutrophils, monocytes and macrophages (Figure 4A, C) and a far denser distribution of cells undergoing apoptotic death (Figure 4B) than those seen in the grafts injected with saline (control of Exp. 8 in Table 2) (Figure 4D–F).

The grafts injected with R2 (Exp. 8 in Table 2) were occupied by connective tissue-like materials containing mononuclear large and small cells and many fibrous cells under thick scab coverings 14 days after the injection (Figure 4G). Dying malignant cells were sporadically discernible, and some of them expressed strong EpoR immunoreactivity (Figure 4G). The control grafts injected with saline (control of Exp. 8 in Table 2) showed well-developed malignant structures with EpoR immunoreactivity (Figure 4H).

### Effects on vasculogenesis

To clarify the dependency of vasculogenesis on Epo signalling in the grafts, we counted the number of cross-and longitudinally sectioned capillaries. The results are shown in Figure 5. The number of capillaries was comparable among the specimens before transplant and in the control grafts with saline injection or needle insertion, but it was significantly lower in the grafts that received injections of R2 or sEpoR ($P < 0.001$).

### DISCUSSION

Withdrawal of Epo signalling in the grafts of female reproductive organ-derived tumours that expressed mRNAs for Epo and EpoR by the local injection of R2 or sEpoR caused marked degeneration of malignant cells and disappearance of capillaries leading to destruction of the tumour masses. The present results

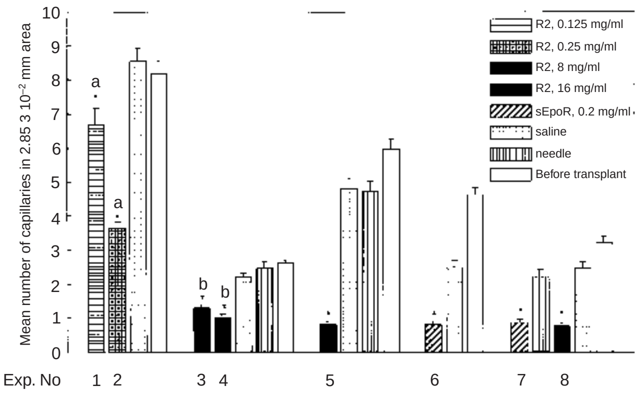

**Figure 5** Average number of capillaries in a definite area of sections of xenografts and original tumours. Counting was done as described in Materials and Methods. Significant differences from controls by Student's *t*-test. *$P < 0.001$. Columns with the same letter show significant differences (a, $P < 0.001$; b, $P < 0.05$)

together with the in vitro findings reported previously strongly suggest that not only proliferation and/or survival of malignant cells in the female reproductive organ-derived grafts but also capillary formation in the grafts, which could be essential for the development of tumour progression, is at least partly attributable to Epo signalling. The present study also revealed the process of elimination of degenerated and dead malignant components by the host's immune and inflammatory cells, leading to repair of the lesion.

The characteristic features of degeneration seen in the tumours were extremely strong expression of EpoR in dying cells, and apoptotic death of the malignant cells. These phenomena were seen in all grafts injected with R2 or sEpoR and sporadically in the degenerating sites of the control grafts. This pattern of tissue degeneration was also detected in the in vitro cultured tumours injected with sEpoR or R2. In the cell line HCD57, the survival of which depends entirely on Epo signalling, EpoR is expressed intensely upon deprivation of Epo (Sawyer et al, 1993). The deprivation of Epo from the culture medium of the cell line appears to reduce the receptor internalization, resulting in a high expression of EpoR in the cytoplasmic membrane. The same is likely to be true for the grafts injected with R2 or sEpoR that decreases the amount of Epo available for cellular EpoR.

Infiltration of a large number of cells including neutrophils, macrophage and monocytes was also seen in the degenerated and amorphous regions of experimental grafts 12 h after the injection. Additionally, many fibrous cells were seen under the scab coverings 7 and 14 days after the treatment. These cells are likely to contribute to the repair of the destroyed regions.

In conclusion, deprivation of Epo signalling caused by the injection of R2 or sEpoR into xenografts of malignant uterine and ovarian tumours induces the destruction of Epo-expressing malignant tumours and capillaries. The present results validated our previous findings that interception of Epo signalling in blocks of cancer specimens from female reproductive organs led to their destruction through the injection of R2 or sEpoR in vitro (in submission). Our results may

 *© 2001 Cancer Research Campaign*

have implications for the treatment of female reproductive cancers characterized by the operation of Epo signal transduction.

### ACKNOWLEDGEMENT

We appreciate Dr Alice S Cary's reading and correction of the manuscript, Hidetoshi Higashiguchi's photographic help, Mie Onozaki's technical assistance, and Keiko Yamashita's aid in preparation of the manuscript.

### REFERENCES

Anagnostou A, Lee ES, Kessimian N, Levinson R and Steiner M (1990) Erythropoietin has a mitogenic and positive chemotactic effect on endothelial cells. *Proc Natl Acad Sci USA* **87**: 5978–5982

Anagnostou A, Liu Z, Steiner M, Chin K, Lee ES, Kessimian N and Noguchi CT (1994) Erythropoietin receptor mRNA expression in human endothelial cells. *Proc Natl Acad Sci USA* **91**: 3974–3978

Bunn HF and Poyton RO (1996) Oxygen sensing and molecular adaptation to hypoxia. *Physiol Rev* **76:** 839–885

Carlini RG, Reyes AA and Rothstein M (1995) Recombinant human erythropoietin stimulates angiogenesis in vitro. *Kidney Int* **47**: 740–745

Goto M, Murakami A, Akai K, Kawanishi G, Ueda M, Chiba H, and Sasaki R (1989) Characterization and use of monoclonal antibodies directed against human erythropoietin that recognize different antigenic determinants. *Blood* **74**: 1415–1423

Guillemin K and Krasnow MA (1997) The hypoxic response: huffing and HIFing. *Cell* **89**: 9–12

Haller H, Christel C, Dannenberg L, Thiele P, Lindschau C and Luft FC (1996) Signal transduction of erythropoietin in endothelial cells. *Kidney Int* **50:** 481–488

Ihle JN (1995) Cytokine receptor signaling. *Nature* **377**: 591–594

Jelkmann W (1992) Erythropoietin: structure, control of production, and function. *Physiol Rev* **72**: 449–489

Komatsu N and Fujita H (1993) Induced megakaryocytic maturation of the human leukemic cell line UT-7 results in down-modulation of erythropoietin receptor gene expression. *Cancer Res* **53**: 1156–1161

Krantz SB (1991) Erythropoietin. *Blood* **77**: 419–434

Lin C-S, Lim S-K, D'Agati V and Costantini F (1996) Differential effects of an erythropoietin receptor gene disruption on primitive and definitive erythropoiesis. *Genes Dev* **10**: 154–164

Masuda S, Nagao M and Sasaki R (1999) Erythropoietic, neurotrophic and angiogenic functions of erythropoietin and regulation of erythropoietin production. *Int J Hematol* **70**: 1–5

Maxwell PH, Pugh CW and Ratcliffe PJ (1993) Inducible operation of the erythropoietin 3′ enhancer in multiple cell lines: Evidence for a widespread oxygen-sensing mechanism. *Proc Natl Acad Sci USA* **90**: 2423–2427

Nagao M, Masuda S, Abe S, Ueda M and Sasaki R (1992) Production and ligand-binding characteristics of the soluble form of murine erythropoietin receptor. *Biochem Biophys Res Commun* **188**: 888–897

Ovejera AA, Houchens DP and Barker AD (1978) Chemotherapy of human tumor xenografts in genetically athymic mice. *Ann Clin Lab Sci* **8**: 50–56

Porter DL and Goldberg MA (1993) Regulation of erythropoietin production. *Exp Hematol* **21**: 399–404

Ratcliffe PJ, O'Rourke JF, Maxwell PH and Pugh CW (1998) Oxygen sensing, hypoxia-inducible factor-1 and the regulation of mammalian gene expression. *J Exp Biol* **201**: 1153–1162

Ribatti D, Presta M, Vacca A, Ria R, Giuliani R, Dell'Era P, Nico B, Roncali L and Dammacco F (1999) Human erythropoietin induces a pro-angiogenic phenotype in cultured endothelial cells and stimulates neovascularization in vivo. *Blood* **93**: 2627–2636

Sadamoto Y, Igase K, Sakanaka M, Sato K, Otsuka H, Sakaki S, Masuda S and Sasaki R (1998) Erythropoietin prevents place navigation disability and cortical infarction in rats with permanent occlusion of the middle cerebral artery. *Biochem Biophys Res Commun* **253**: 26–32

Sakanaka M, Wen T-C, Matsuda S, Masuda S, Morishita E, Nagao M and Sasaki R (1998) In vivo evidence that erythropoietin protects neurons from ischemic damage. *Proc Natl Acad Sci USA* **95**: 4635–4640

Sawyer ST and Hankins WD (1993) The functional form of the erythropoietin receptor is a 78-kDa protein: Correlation with cell surface expression, endocytosis, and phosphorylation. *Proc Natl Acad Sci USA* **90**: 6849–6853

Semenza GL (1998) Hypoxia-inducible factor 1 and the molecular physiology of oxygen homeostasis. *J Lab Clin Med* **131**: 207–214

Wenger RH and Gassmann M (1997) Oxygen (es) and the hypoxia-inducible factor-1. *Biol Chem* **378**: 609–616

Wu H, Liu X, Jaenisch R and Lodish HF (1995) Generation of committed erythroid BFU-E and CFU-E progenitors does not require erythropoietin or the erythropoietin receptor. *Cell* **83**: 59–67

Yamaji R, Okada T, Moriya M, Naito M, Tsuruo T, Miyatake K and Nakano Y (1996) Brain capillary endothelial cells express two forms of erythropoietin receptor mRNA. *Eur J Biochem* **239**: 494–500

Yasuda Y, Nishi N, Takahashi JA, Konishi H, Ohara I, Fujita H, Ohta M, Itoh N, Hatanaka M and Tanimura T (1992) Induction of avascular yolk sac due to reduction of basic fibroblast growth factor by retinoic acid in mice. *Dev Biol* **150**: 397–413

Yasuda Y, Nagao M, Okano M, Masuda S, Sasaki R, Konishi H and Tanimura T (1993) Localization of erythropoietin and erythropoietin receptor in postimplantation mouse embryos. *Develop Growth Differ* **35**: 711–721

Yasuda Y, Masuda S, Chikuma M, Inoue K, Nagao M and Sasaki R (1998) Estrogen-dependent production of erythropoietin in uterus and its implication in uterine angiogenesis. *J Biol Chem* **273**: 25381–25387

Youssoufian H, Longmore G, Neumann D, Yoshimura A and Lodish HF (1993) Structure, function, and activation of the erythropoietin receptor. *Blood* **81**: 2223–2236

© 2001 Cancer Research Campaign 
